# Targeting age‐specific changes in CD4^+^ T cell metabolism ameliorates alloimmune responses and prolongs graft survival

**DOI:** 10.1111/acel.13299

**Published:** 2021-01-26

**Authors:** Yeqi Nian, Jasper Iske, Ryoichi Maenosono, Koichiro Minami, Timm Heinbokel, Markus Quante, Yang Liu, Haruhito Azuma, Jinrui Yang, Reza Abdi, Hao Zhou, Abdallah Elkhal, Stefan G. Tullius

**Affiliations:** ^1^ Division of Transplant Surgery and Transplant Surgery Research Laboratory Brigham and Women's Hospital Harvard Medical School Boston MA USA; ^2^ Department of Urology Second Xiangya Hospital Central South University Changsha China; ^3^ Department of Kidney Transplantation Tianjin First Central Hospital Nankai University Tianjin China; ^4^ Institute of Transplant Immunology Hannover Medical School Hannover Germany; ^5^ Department of Urology Osaka Medical College Osaka Japan; ^6^ Department of Pathology Charité – Universitätsmedizin Berlin Berlin Germany; ^7^ Department of General, Visceral‐ and Transplant Surgery University Hospital Tübingen Tubingen Germany; ^8^ Institute of Hepatobiliary Diseases Zhongnan Hospital of Wuhan University Wuhan China; ^9^ Renal Division Transplantation Research Center Brigham and Women's Hospital Harvard Medical School Boston MA USA

**Keywords:** aging, cellular immunology, cellular senescence, interleukin 2, metabolic rate, mitochondria, respiratory chains, T cell

## Abstract

Age impacts alloimmunity. Effects of aging on T‐cell metabolism and the potential to interfere with immunosuppressants have not been explored yet. Here, we dissected metabolic pathways of CD4^+^ and CD8^+^ T cells in aging and offer novel immunosuppressive targets. Upon activation, CD4^+^ T cells from old mice failed to exhibit adequate metabolic reprogramming resulting into compromised metabolic pathways, including oxidative phosphorylation (OXPHOS) and glycolysis. Comparable results were also observed in elderly human patients. Although glutaminolysis remained the dominant and age‐independent source of mitochondria for activated CD4^+^ T cells, old but not young CD4^+^ T cells relied heavily on glutaminolysis. Treating young and old murine and human CD4^+^ T cells with 6‐diazo‐5‐oxo‐l‐norleucine (DON), a glutaminolysis inhibitor resulted in significantly reduced IFN‐γ production and compromised proliferative capacities specifically of old CD4^+^ T cells. Of translational relevance, old and young mice that had been transplanted with fully mismatched skin grafts and treated with DON demonstrated dampened Th1‐ and Th17‐driven alloimmune responses. Moreover, DON diminished cytokine production and proliferation of old CD4^+^ T cells in vivo leading to a significantly prolonged allograft survival specifically in old recipients. Graft prolongation in young animals, in contrast, was only achieved when DON was applied in combination with an inhibition of glycolysis (2‐deoxy‐d‐glucose, 2‐DG) and OXPHOS (metformin), two alternative metabolic pathways. Notably, metabolic treatment had not been linked to toxicities. Remarkably, immunosuppressive capacities of DON were specific to CD4^+^ T cells as adoptively transferred young CD4^+^ T cells prevented immunosuppressive capacities of DON on allograft survival in old recipients. Depletion of CD8^+^ T cells did not alter transplant outcomes in either young or old recipients. Taken together, our data introduce an age‐specific metabolic reprogramming of CD4^+^ T cells. Targeting those pathways offers novel and age‐specific approaches for immunosuppression.

## Graphical abstract

Upon activation, old CD4^+^ T cells failed to exhibit adequate metabolic reprogramming resulting into compromised metabolic pathways. Since glutaminolysis displayed their dominant fuel source for mitochondria, treatment with a glutaminolysis inhibitor (DON) restricted activation, proliferative capacities and cytokine production in an age‐specific manner. These effects translated into dampened alloimmune responses and dramatically prolonged allograft survival specifically in old transplant recipients.

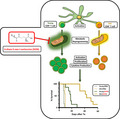

## 1 INTRODUCTION

Immunosenescence impacts T‐cell alloimmunity broadly. While memory T cells accumulate in aging, old T cells are less effective and show compromised proliferative capacities in addition to an impaired Th1 immunity (Bedi et al., 2016; Colvin et al., 2017). Of clinical relevance, established immunosuppressants demonstrate age‐specific effects. We have previously linked a compromised interleukin‐2 (IL‐2) production in old human and murine CD4^+^ T cells to an impaired calcium metabolism and a more effective immunosuppression with Tacrolimus (Krenzien et al., 2017).

Metabolic reprogramming is critical for the activation, proliferation, and differentiation of CD4^+^ T cells (Ricciardi et al., 2018). Upon activation, T‐cell receptor (TCR) signaling guides the metabolization of glucose to aerobic glycolysis; moreover, an amplified glutaminolysis results into an increased production of glutamate, fueling mitochondria (Kouidhi et al., 2017). These metabolic effects enable CD4^+^ T cells to rapidly generate a large amount of ATP through aerobic glycolysis while maintaining mitochondrial function. With those adaptations, T cells respond to the high demand of energy necessary for proliferation and differentiation (Kouidhi et al., 2017).

Following allogeneic organ transplantation, T‐cell‐driven alloimmune responses are initiated in response to major histocompatibility complex (MHC) differences between donor and recipient (DeWolf & Sykes, 2017). Subsequently, antigen‐presenting cells including dendritic cells present donor antigens via MHC‐II molecules that activate naïve T cells, augmenting their proliferation and pro‐inflammatory cytokine production (Alegre et al., 2016). Metabolic reprogramming plays a vital role for T‐cell activation. Targeting T‐cell metabolism may therefore constitute an innovative immunosuppressive approach. Notably, blocking the metabolic reprogramming of alloreactive T cells through a cocktail of three metabolic inhibitors has recently been shown to exert significant immunosuppressive capacities by suppressing the CD4^+^ T‐cell compartment in young mice (Lee et al., 2015).

With a rapidly growing number of elderly patients that are more susceptible to side effects of established immunosuppressants, age‐specific characteristics of alloimmunity have been investigated (Iske et al., 2020; Oberhuber et al., 2015). How aging alters metabolic pathways of T cells and the relevance of this process for T‐cell‐driven alloimmune responses has not been delineated. Moreover, it remains unclear if age‐dependent T‐cell metabolism will provide novel immunosuppressive approaches. Recently, we have described an age‐specific prolongation of graft survival utilizing tacrolimus or rapamycin (Krenzien et al., 2017; Quante et al., 2018), two immunosuppressive drugs impairing the metabolic function of T cells through the inhibition of the mTOR pathway (Lombardi et al., 2017).

To explore the impact of aging on metabolic pathways in more detail, we performed extracellular flux analyses and mitochondrial‐stress assays delineating metabolic phenotypes of young and old CD4^+^ T cells (3 and 18 mths, respectively). This approach characterized a diminished metabolic capacity of old CD4^+^ T cells with a compromised OXPHOS and glycolytic activity. Moreover, we observed that the respiratory capacity of CD4^+^ T cells had been reduced age specifically. Upon administration of metabolic inhibitors targeting the mitochondrial electron transport chain, old but not young mouse and human CD4^+^ T cells failed to compensate the restrained OXPHOS‐derived energy generation through the augmentation of glycolysis. Of additional relevance, glutaminolysis demonstrated to be cardinal source fueling the OXPHOS pathway upon T‐cell activation in both old and young T cells. These observations translated into a dose‐dependent mitigation of proliferation and cytokine production in old but not young CD4^+^ T cells when inhibiting glutaminolysis. As an underlying mechanism, we identified a pronounced inhibition of the mTOR pathway with a downregulated phosphorylation of S6 ribosomal protein (pS6) in addition to a reduced expression of c‐Myc in old CD4^+^ T cells.

As a consequence, allograft survival was significantly prolonged in old recipient mice when inhibiting glutaminolysis. In young animals, comparable graft prolongation was only achieved with a combinatorial treatment inhibiting glutaminolysis in addition to restraining glycolytic and OXPHOS pathways.

Collectively, our studies introduce an age‐specific immunosuppression targeting metabolic pathways of CD4^+^ T cells.

## 2 RESULTS

### 2.1 Old CD4^+^ T cells exhibit compromised OXPHOS and glycolysis pathways upon activation

Immunosenescence is linked to a broad range of quantitative and qualitative changes impacting several cellular subpopulations. The most striking alterations have been found within the T‐cell compartment with compromised T‐cell receptor signaling, distinct proliferative limitations, and altered cytokine expression following activation (Bektas et al., 2017; Heinbokel et al., 2013). The impact of immunosenescence on T‐cell metabolism has thus far not been delineated. We thus started our experimental series by investigating age‐specific metabolic characteristics of naïve CD4^+^ T cells following TCR activation. In detail, we isolated naïve CD4^+^ T cells from 3‐ and 18‐mth‐old mice activated with 10 µg/ml anti‐CD3 and 2 µg/ml anti‐CD28. After 24 h of activation, we made use of a Mito Stress assay using the Seahorse XFe96 extracellular flux analyzer to evaluate age‐specific changes in CD4^+^ T‐cell metabolism. Oxygen consumption rate (OCR) reflecting oxidative phosphorylation (OXPHOS) in mitochondria and extracellular acidification rate (ECAR) indicates glycolysis activity in cells (Zhang & Zhang, 2019).

**FIGURE 1:**
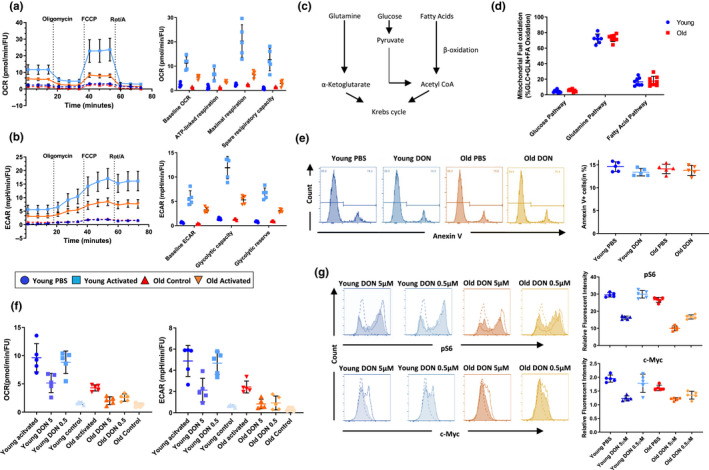
Inhibiting glutaminolysis modifies CD4^+^ T‐cell metabolism age specifically. (a and b) Naïve CD4^+^ T cells were isolated from young and old C57BL/6 and activated with 10 µg/ml anti‐CD3 and 2 µg/ml soluble antiCD28 for 24 h. Control groups were maintained in IL‐2 (50 ng/ml). OCR, ECAR, and calculated metabolic parameters utilizing a Seahorse Mito Stress assay are shown. (c) Mitochondrial fueling pathways. (d) Dependence for mitochondrial fuel on distinct metabolic pathways of young and old naïve CD4^+^ T cells 24 h of activation. (e) Naïve CD4^+^ T cells from young and old mice were activated with anti‐CD3/anti‐CD28 under the condition of 5 μM DON or PBS. Cell death was measured by the frequency of Annexin V^+^ cells. (f) OCR and ECAR of young and old naïve, unspecifically activated CD4^+^ T cells were measured by 24 h following the addition of DON (5 μM, 0.5 μM, or PBS). Control group as maintained in 50 ng/ml IL‐2. (g) The phosphorylation state of the S6 ribosomal protein and c‐Myc expression were measured in young and old naïve CD4^+^ T cells in the presence of DON (5 μM, 0.5 μM) or PBS. Column plots display individual data points and mean ± *SD*, *n* = 5/group. The results are representative of at least three independent experiments

Our results showed that activated old naïve CD4^+^ T cells had a significantly compromised activity of mitochondrial OXPHOS (Figure 1a). Moreover, lower basic ECAR levels after activation suggested a compromised glycolysis in old CD4^+^ T cells (Figure 1b). In addition, we observed a significantly reduced ATP linked respiration and maximal respiratory capacity in old naïve CD4^+^ T cells (Figure 1a). Furthermore, old naïve CD4^+^ T cells demonstrated a significantly compromised spare respiratory capacity measured under conditions mimicking metabolic stress (*p* < 0.01; Figure 1a). These results suggested a compromised mitochondrial activity of old naïve CD4^+^ T cells and a limited capacity of mitochondrial OXPHOS in response to metabolic stressors.

With a compromised mitochondrial ATP production, we reasoned that naïve old CD4^+^ T cells may augment aerobic glycolysis as a compensatory response. Interestingly, old but not young naïve CD4^+^ T cells were unable to compensate their metabolic disadvantage with an augmented glycolysis when mitochondrial‐derived ATP generation had been completely inhibited through oligomycin and FCCP (Figure 1b).

T cells utilize glucose, glutamine, and fatty acids to fuel their mitochondria (van der Windt & Pearce, 2012; Figure 1c). During quiescence, T cells metabolize glucose to generate energy while glutamine takes the place of glucose to maintain mitochondrial function when T cells become activated. Our data showed that T‐cell aging modifies mitochondrial function and glycolysis. Since changes in the metabolic pathway may also affect the selection of fuel by mitochondria, (Cortassa et al., 2019), we next dissected the energy source utilized by mitochondria in young and old naïve CD4^+^ T cells using a mito fuel assay.

Notably, in both young and old CD4^+^ T cells, glutaminolysis appeared as the dominant pathway of energy recruitment. Moreover, the utilization of glucose as fuel of mitochondria in activated CD4^+^ T cells did not change in aging (Figure 1d).

Taken together, our results demonstrated a diminished OXPHOS and glycolysis activity of old naïve CD4^+^ T cells upon activation while glutaminolysis remained the major source of energy. More importantly, when the generation of ATP by mitochondria was inhibited, old CD4^+^ T cells had been unable to compensate for their metabolic limitations.

### 2.2 Inhibition of glutaminolysis exhibits age‐specific effects on metabolic reprogramming

To evaluate whether an inhibition of glutaminolysis would exert toxic side effects on CD4^+^ T cells, we evaluated the viability of young and old naïve CD4^+^ T cells following activation via their TCR in the presence of 5 μM DON or PBS. By 24 h, the rate of apoptosis was determined by assessing the frequency of Annexin V^+^ cells. As shown in Figure 1e, young and old naïve CD4^+^ T cells demonstrated a comparable cell viability. Moreover, the application of 5 µM DON did not increase the rate of apoptosis for old naive CD4^+^ T cells.

Next, we tested the effects of inhibiting glutaminolysis by DON on the metabolic functionality of either young or old naïve CD4^+^ T cells using the extracellular flux assay. As shown in Figure 1f, high concentrations of DON (5 µM) inhibited OCR and ECAR significantly in both, young and old naïve CD4^+^ T cells (DON vs. PBS, young: *p* < 0.01, old: *p* < 0.001). More importantly, when DON was administered at a lower dose (0.5 µM), OCR and ECAR had only been reduced in old but not young naïve CD4^+^ T cells (*p* > 0.05; Figure 1f). Notably, DON (0.5 µM) did not impact metabolic parameters of young naïve CD4^+^ T cells. In contrast, old naïve CD4^+^ T cells treated with either high (5 µM) or low (0.5 µM) DON concentrations demonstrated comparable ECAR with elevated OCR levels (*p* < 0.05; Figure 1f), indicating that metabolic reprogramming had been specifically relevant for old CD4^+^ T cells. Notably, young CD4^+^ T cells showed higher OCR and ECAR rates subsequent to the application of both, high‐dose (5 µM) or low‐dose (0.5 µM) DON (Figure S1A).

It is well established that naïve CD4^+^ T cells exhibit an increased activation of mTOR pathway via Akt phosphorylation and Raptor phosphorylation through TCR/CD28 upon activation (Han et al., 2012; Liu et al., 2015). Moreover, mTOR activation has been shown to promote metabolic reprogramming through the up‐regulation of key transcriptional factors including c‐Myc, HIF‐1α, and IRF4 (Waickman & Powell, 2012). Importantly, elevated c‐Myc‐mediated cellular metabolic reprogramming has been shown to augment nutrition transporters in addition to key enzymes involved in glycolysis and glutaminolysis (Wang et al., 2011).

To evaluate whether DON may exert its age‐specific effects on metabolic reprogramming through altering the mTOR signaling pathway, we measured phosphorylate S6 (pS6), as an established marker of mTOR signaling (Leontieva et al., 2012). Moreover, we assessed the consequences of DON on the key transcriptional factor c‐Myc downstream of mTOR signaling. As shown in Figure 1g, 0.5 µM DON inhibited pS6 specifically in old but not young naïve CD4^+^ T cells (*p* < 0.001). Consistent with these data, low‐dose DON demonstrated an age‐specific inhibition of c‐Myc expression as confirmed by FACS and RT‐PCR (*p* < 0.05; Figure 1g and Figure S1B).

### 2.3 Glutaminolysis plays a central role for activation, cytokine production, and proliferation of old CD4^+^ T cells

**FIGURE 2:**
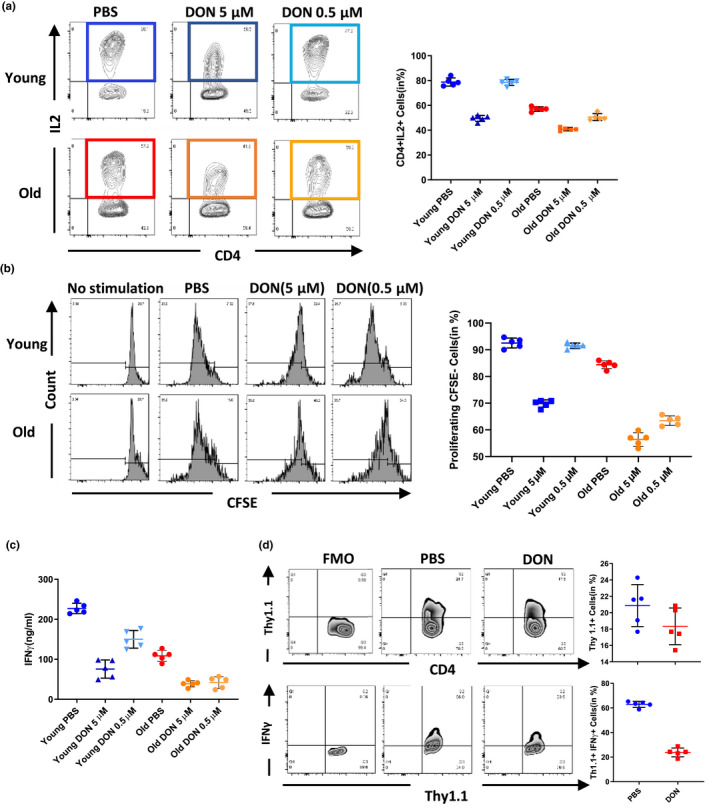
DON inhibited CD4^+^ T‐cell immunity age specifically in vitro and in vivo. (a) IL‐2 production of young and old naïve CD4^+^ T cells was measured 24 h after unspecific activation (10 µg/ml anti‐CD3 and 2 µg/ml anti‐CD28). (b) Naïve CD4^+^ T cells were isolated from young and old C57BL/6 mice and labeled with CFSE. Proliferation of naïve CD4^+^ T cells was measured by the dilution of CFSE, after 72 h in co‐cultures with DBA‐derived splenocytes. (c) IFN‐γ was measured by ELISA in cultural supernatants. (d) OT‐II mice‐derived CD4^+^ T cells were adoptively transferred into wild‐type C57BL/6 mice. Recipients were then stimulated with 500 μg OVA. Proliferation and function of OT‐II CD4^+^ T cells were measured by flow cytometry (day 3). Column plots display individual data points and mean ± *SD*, *n* = 5/group. The results are representative of at least three independent experiments

Metabolic reprogramming has been shown to constitute a crucial pathway for CD4^+^ T‐cell activation and proliferation (Ganeshan & Chawla, 2014). To dissect age‐specific effects of glutamine inhibition on CD4^+^ T cells, we next evaluated the expression of IL‐2 (Busse et al., 2010), a well‐established marker for CD4^+^ T activation. Those experiments were performed in young and old naïve CD4^+^ T cells stimulated with anti‐CD3/anti‐CD28 following DON treatment. Notably, DON (5 µM) inhibited the expression of IL‐2 in both, young and old naive CD4^+^ T cells. At a lower dose (0.5 µM), however, DON specifically inhibited IL‐2 expression only in old naïve CD4^+^ T cells (Figure 2a), suggesting dose‐dependent and age‐specific effects of DON.

To test for a potential immunosuppressive role of DON, we next performed mixed lymphocyte reactions (MLR) dissecting CD4^+^ T‐cell‐driven alloimmune responses. After transplanting young and old C57BL6 mice with fully mismatched skin allografts (DBA2/J donors), CD4^+^ splenocytes were isolated and proliferation was assessed by carboxyfluorescein succinimidyl ester (CFSE) staining. Our findings indicated that low doses of DON (0.5 µM) inhibited the proliferation of old CD4^+^ T cells specifically (Figure S2B). More importantly, high doses of DON (5 µM) reduced levels of IFNγ production in both, old and young CD4^+^ T cells; lower doses of DON (0.5 µM) decreased IFNγ production only in old CD4^+^ T cells (*p* < 0.01; Figure S2C).

These data suggest that activation, pro‐inflammatory cytokine production, and proliferation of old CD4^+^ T cells can be inhibited with low dose of DON. Those observations also confirmed the unique role of glutaminolysis in old CD4 T cells.

### 2.4 Glutaminolysis regulates CD4^+^ T‐cell‐driven alloimmune responses in old recipients

Next, we evaluated the potential of DON to inhibit antigen‐specific CD4^+^ T‐cell responses in OT‐II transgenic mice. OT‐II transgenic mice express the mouse α‐ and β‐chain T‐cell receptor that pairs with the CD4 co‐receptor, specific for chicken ovalbumin (OVA) 323–339 peptide in the context of I‐Ab. Challenging OT‐II mice with OVA specifically activates OT‐II CD4^+^ T cells in an MHC‐II‐dependent manner. This model allows to delineate CD4^+^‐specific alloimmune responses to MHC class II (Barnden et al., 1998).

OT‐II CD4^+^ T cells (on a C57BL/6 background) were isolated and adoptively transferred into C57BL/6 mice (3 months) that were challenged with 500 µg OVA; recipient animals were subsequently treated with injections of 1.6 mg/kg DON or PBS every other day. In line with our in vitro data, mice treated with DON contained significantly less IFN^+^Thy1.1^+^T cells (*p* < 0.001, Figure 2d) while amounts of CD4^+^Thy1.1^+^T cells remained unchanged (Figure 2d), indicating that DON inhibited OVA‐specific effector functions without impairing proliferation.

**FIGURE 3:**
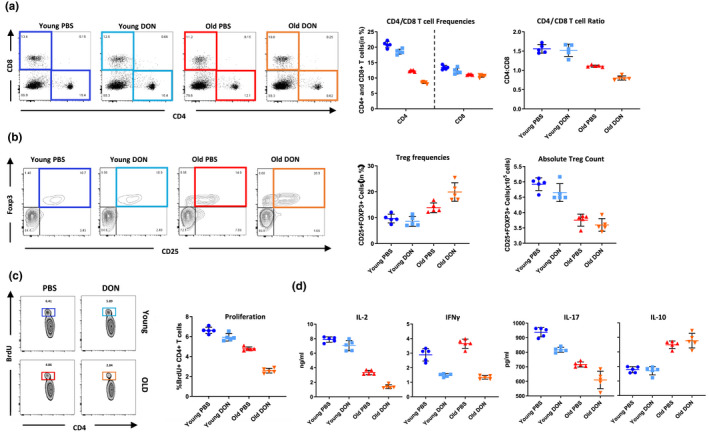
DON dampens alloimmune responses in vivo in an age‐specific manner. Skin allografts from young DBA mice were transplanted onto young and old C57BL/6 mice. Recipients were treated with 1.6 mg/kg DON q.o.d or PBS starting on the day of transplantation. (a) Frequencies of CD4^+^ and CD8^+^ T cells and (b) frequencies and absolute numbers of Tregs in the spleen were assessed after 7 days by flow cytometry gating on single cells. (c) Recipient mice received a single intraperitoneal injection of BrdU prior to skin engraftment; animals were then treated with DON or PBS. 7 days after transplantation, splenic frequencies of BrdU^+^ cells were analyzed by flow cytometry. (d) Splenocytes from young and old C57BL/6 recipients of DBA skin allografts were collected 7 days after transplantation. Recipient mice received DON or PBS. Splenocytes were co‐cultured with anergic DBA splenocytes. By 48 h, cytokine secretion was measured by ELISA. Column plots display individual data points and mean ± *SD*, *n* = 5/group. The results are representative of at least three independent experiments

As the OT‐II TCR transgenic mouse model is immunodeficient and has as such a limited life span (Leung et al., 2013), we next dissected age‐specific effects of DON on T‐cell metabolism, in vivo, using a fully MHC mismatched skin transplantation model. Young and old C57BL6 mice that had received skin allografts from allogeneic DBA2/J donors were treated with injections of DON or PBS every other day. After day 7, alloimmune responses of CD4^+^ T cells were evaluated by flow cytometry analysis. DON treatment reduced frequencies of CD4^+^ T‐cell subsets in both, young and old recipients; CD8^+^ frequencies, in contrast, remained unchanged (Figure 3a). Moreover, old animals treated with DON showed a reversed CD4^+^/CD8^+^ ratio, suggesting that old CD4^+^ T cells had been specifically prone to DON treatment (Figure 3a). Interestingly, frequencies of regulatory T cells, that have been shown to exert prominent immunosuppressive effects on alloreactive T cells (Scalea et al., 2016), increased specifically in old recipients after DON treatment (Figure 3b). It is important to note that DON also reduced the total number of CD4^+^ T cells more prominently in old mice, while absolute Treg numbers remained unchanged following DON treatment (Figure 3b), indicating limited effects of DON treatment on the Treg subset (Figure 3a).

Next, we assessed the proliferation of CD4^+^ T cells in our skin transplant model utilizing a BrdU assay. Consistent with our in vitro data, DON inhibited the proliferation of CD4^+^ T cells specifically in old mice (Figure 3c). To further dissect age‐specific immunosuppressive effects of DON on CD4^+^ T‐cell‐driven alloimmune responses, we re‐stimulated both, young and old CD4^+^ T cells from skin graft recipients with donor‐derived splenocytes in MLR experiments and assessed the production of cytokines by ELISA. Consistent with our previous results, old‐ and young‐treated CD4^+^ T cells exhibited compromised levels of IL‐2, IFNγ, and IL‐17, while IL‐10, a cytokine produced by Tregs, remained unchanged following DON administration (Figure 3d).

Overall, these results indicated that the inhibition of glutaminolysis through DON exerts potent immunosuppressive effects on alloimmune responses, suggesting that the relatively increased frequency of Tregs in old recipients may contribute to the observed age‐specific allograft survival.

### 2.5 Age‐specific immunosuppressive effects of DON are not mediated through CTPS1 inhibition

Notably, DON has also been shown to inhibit the CTP synthase 1 (CTPS1), an enzyme catalyzing the final committed step of the pyrimidine biosynthesis (Kassel et al., 2010). It has been shown that CTPS1 is decisively involved in promoting the proliferation of T cells following their activation. T cells from patients displaying a CTPS1‐deficiency have been shown to lack the capacity to sustain proliferative responses and adequate IL‐2 production in response to TCR activation (Martin et al., 2014, 2020). To delineate, if DON exerts the observed age‐specific immunosuppressive effects through inhibiting CTPS1, we made use of an alternative way to block the glutaminolysis pathway by using CB‐839, a specific inhibitor of the phosphate‐activated mitochondrial glutaminase (GLS1) (Gross et al., 2014). Notably, CB‐839 treatment exhibited comparable effects on T‐cell metabolism and alloimmune responses as DON with compromised spare respiratory capacity (Figure S2A) and glycolytic reserve (Figure S2B) while dampening proliferation (Figure S2D) and IFN‐γ production (Figure S2E) specifically in old CD4^+^ T cells. Of note, c‐myc expression had been diminished in both young and old CD4^+^ T cells (Figure S2C).

Those results support our findings that glutaminolysis constitutes the major target of DON in mediating its age‐specific immunosuppressive effects.

### 2.6 DON prolongs graft survival age specifically

**FIGURE 4:**
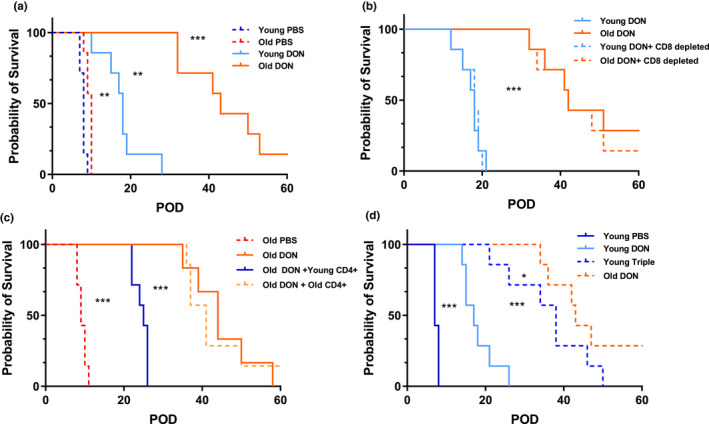
DON prolongs graft survival in old recipients by targeting 7CD4^+^ T‐cell metabolism. (a) Skin allografts from young DBA mice were transplanted onto young and old C57BL/6 mice. Recipients were treated with 1.6 mg/kg DON g.o.d. or PBS starting on the day of transplantation. Graft survival was monitored daily over 60 days. (b) CD8^+^ T cells of recipient mice were depleted with anti‐CD8 monoclonal antibody. Graft survival was recorded and compared to wild‐type C57BL/6 mice. (c) CD4^+^ T cells isolated from young or old C57BL/6 mice were adoptively transferred to old recipients and graft survival monitored after DON or PBS treatment. (d) Fully mismatched young skin transplant recipients were treated with triple treatment (DON, 2‐DG and metformin), or PBS. An additional group of old recipients was treated with DON only. Statistical significance for survival data was determined by log‐rank Mantel–Cox test, *n* = 7/group. Asterisks indicate *p*‐values *p ≤ 0.05, **p ≤ 0.01, and ***p ≤ 0.001, only significant values are shown

To test the translational relevance of our findings, we next grafted old and young C57BL/6 mice with allogeneic skin from DBA2/J donor mice. Notably, recipients treated with 1.6 mg/kg DON (i.p., q.a.d.) demonstrated significantly prolonged survival times of skin allografts that were specifically pronounced in old animals (Figure 4a), emphasizing on the reliance of old T cells on glutaminolysis.

### 2.7 DON prolongs graft survival specifically mediated by CD4^+^ T cells

Our previous work has shown that age‐specific effects of established immunosuppressants were predominantly mediated by CD4^+^ T cells (Krenzien et al., 2017; Quante et al., 2018). Thus, to specifically dissect whether glutaminolysis plays a specific role in alloimmune responses driven by CD4^+^ T cells, we selectively depleted CD8^+^ T cells in recipient animals with daily i.p. injections of an anti‐CD8 monoclonal antibody. Notably, the depletion of CD8^+^ T cell did not impact graft survival (Figure 4b), suggesting that the glutaminolysis pathway plays a key role in an age‐specific alloimmune response mediated via CD4^+^ but not CD8^+^ T cells.

To further confirm the link between age‐specific immunosuppressive effects communicated by DON and the observed compromised metabolism in old CD4^+^ T cells, young or old CD4^+^ T cells were adoptively transferred into old recipients. Remarkably, adoptive transfer of young CD4^+^ T cells prevented the effects of DON in prolonging graft survival in an age‐specific fashion, confirming that glutaminolysis inhibition is less effective in young CD4^+^ T cells (Figure 4c).

Finally, we extended our metabolic treatment not only by inhibiting glutaminolysis (through the application of DON) but also by inhibiting glycolysis (through 2‐DG) and the mitochondrial electron transport chain (through metformin). Consistent with previous reports (Lee et al., 2015), triple treatment prolonged allograft survival in young recipients. Notably, prolongation of allograft survival under triple treatment in young recipients remained inferior to the application of DON only in promoting graft survival in old recipient animals (Figure 4d).

### 2.8 DON inhibits human CD4^+^ T‐cell age specifically

**FIGURE 5:**
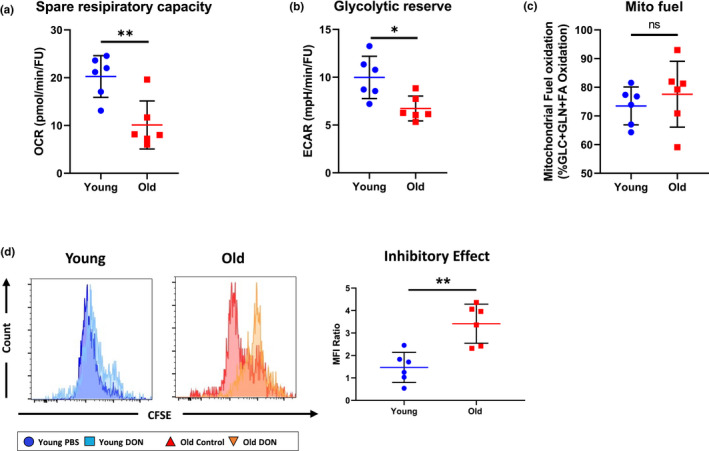
DON inhibits human CD4^+^ T‐cell age specifically. (a and b) Naïve human CD4^+^ T cells were isolated from young and old healthy volunteers and activated with 10 µg/ml anti‐CD3 and 2 µg/ml soluble anti‐CD28 for 24 h. Calculated spare respiratory capacity and glycolytic reserve were assessed utilizing a Seahorse Mito Stress. (c) Dependence for mitochondrial fuel on distinct metabolic pathways of young and old naïve human CD4^+^ T cells 24 h of activation is shown. (d) Naïve CD4^+^ T cells were isolated from young and old healthy volunteers, cultured in a mixed lymphocyte reaction to which either PBS or DON had been added and labeled with CFSE. After 72 h, proliferation of naïve CD4^+^ T cells was measured by the dilution of CFSE and the inhibitory effects of DON measured by the ratio of CFSE median fluorescence intensity between the DON and the PBS group. Column plots display individual data points and mean ± *SD*, *n* = 5/group. Statistical significance was determined by using Mann–Whitney test. Asterisks indicate *p*‐values *p ≤ 0.05, **p ≤ 0.01, and ***p ≤ 0.001, only significant values are shown. The results are representative of at least three independent experiments

To evaluate the translational relevance of our findings, we next set out to confirm our experimental results in human CD4^+^ T cells. Therefore, human CD4^+^ T cells were purified from PBMCs of young (<30 years) and old (>65 years) healthy volunteers and the metabolic performance was assessed with a Seahorse Mito Stress assay. Consistent with our experimental data, old human CD4^+^ T cells exhibited a compromised spare respiratory capacity and an inferior glycolytic reserve when compared to CD4^+^ T cells derived from young donors (Figure 5a,b). Moreover, glutaminolysis displayed the dominant fuel source in activated human CD4^+^ T cells regardless of aging (Figure 5c).

Based on these results, we next treated young and old human CD4^+^ T cells with 0.5 µM DON or PBS and performed mixed lymphocyte reactions assessing their proliferative capacities by CFSE staining. Consistent with the effect of DON on murine CD4^+^ T cells, 0.5 µM DON also inhibited the proliferation of old but not young CD4^+^ T cells (Figure 5d).

## 3 DISCUSSION

With rapid demographic changes, elderly patients are transplanted at increasing rates. Indeed, more than 60% of all transplant recipients are currently older than 50 years and more than 20% of all organ transplant recipients being older than 65 years. Although advantages of transplantation have been established for elderly recipients (Colvin et al., 2017), side effects of immunosuppressants including infections or malignancies are particularly detrimental in older recipients (Krenzien et al., 2015). Notably, the most frequent cause of death in older transplant recipients is linked to immunosuppressive side effects (Krenzien et al., 2015). Thus, age‐specific immunosuppressive regimens appear in need. Age‐adapted immunosuppression, however, should not necessarily be understood as a dose reduction in established immunosuppressants but rather as a specific selection and application of immunosuppressants in the elderly. We have previously shown in experimental and clinical studies that tacrolimus, a calcineurin inhibitor, is more effective in older recipients linked to age‐specific calcineurin levels (Krenzien et al., 2017). Moreover, we have demonstrated that the mTOR inhibitor Rapamycin promoted allograft survival in older recipients by exerting age‐specific effects that were linked to the induction of CD4^+^IFNγ^+^IL‐10^+^ regulatory type 1 cells (Quante et al., 2018).

Our current study demonstrates age‐specific aspects of T‐cell metabolism in alloimmunity. Of particular relevance, age‐specific metabolization rates were targetable and prolonged skin graft survival specifically in old recipients. Indeed, we were able to show that old but not young CD4^+^ T cells have limited compensatory capacities to respond to an inhibition of glutaminolysis. Specifically, DON blocked metabolic reprogramming and inhibited activation and proliferation of naïve CD4^+^ T cells in an age‐specific fashion. Our studies have been built on previous findings, showing that interfering with T‐cell metabolism has immunosuppressive capacities (Lee et al., 2015). Here, we show that aging results in profound metabolic changes and demonstrate that glutaminolysis is a critical pathway in the aging T‐cell alloimmune responses.

Glutaminolysis has been shown to activate the mTOR signaling machinery (Duran et al., 2012). Moreover, tacrolimus (FK506) regulates intracellular calcium release and has also been suggested to interfere with the glutaminolysis pathway (Chung et al., 2018; Miller et al., 2018). Both rapamycin and tacrolimus are known to competitively interact with FK506‐binding protein‐12 (FKBP12) (Kim & Guan, 2019). Our current findings are therefore consistent with our previous reports, suggesting that both tacrolimus and rapamycin prolong allograft survival specifically in older recipients, at least in part through interfering with the glutaminolysis pathway.

Our current data also show that age‐specific effects of DON were predominantly mediated by CD4^+^ T cells. This observation is also supported by our own previous work showing the critical role of CD4^+^ T cells in age‐specific alloimmune responses (Krenzien et al., 2017; Quante et al., 2018). DON led to a decreased CD4^+^ T‐cell frequency while not impacting CD8^+^ T‐cell counts. An age‐specific decline of the CD4/CD8 ratio, however, indicated a more robust inhibition of DON in old CD4^+^ T cells. Consistent with these results, DON inhibited the proliferation of CD4^+^ T cell in vivo in an age‐specific manner. Our data also confirm a specific role of CD4^+^ T‐cell responses in aging that remained evident in the absence of CD8^+^ T cells. Moreover, adoptively transferred young CD4^+^ T cells reversed age‐specific effects of DON in old recipients. The translational potential of our experimental data has also been confirmed in human CD4^+^ T cells. Consistent with our experimental findings, we observed a compromised metabolic reserve of old human CD4^+^ T cells. Moreover, the proliferative capacity of old human CD4^+^ T cells was inhibited by low‐dose DON.

Metabolic interventions have emerged as novel and promising approaches to regulate immune responses. Based on the critical role of metabolic reprogramming during T‐cell activation and differentiation, targeting T‐cell metabolism has been efficient in treating diseases mediated by T‐cell immunity (Ganeshan & Chawla, 2014). In a murine lupus model, for example, blocking glycolysis reduced the production of IFN‐γ and IL‐17 and prevented disease progress (Yin et al., 2016). The relevance of age‐specific modifications in T‐cell metabolism is also supported by work showing a distinct calcium metabolism in old CD4^+^ T cells regulated by glycolysis and oxidative phosphorylation (Fracchia et al., 2013; Krenzien et al., 2017; Vaeth et al., 2017). Those aspects may make the immunosuppressive capacity of CNIs in elderly transplant recipients with reduced Calcineurin levels more effective.

The amplification of mitochondrial energy supply is essential for an effective CD4^+^ T cells activation (Ganeshan & Chawla, 2014). Upon activation, CD4^+^ T cells enhance glutaminolysis and recruit α‐KG as the major mitochondrial fuel. Although glycolysis has been compromised in old CD4^+^ T cells, our data show that the metabolism of mitochondria relies largely on glutaminolysis without switching to glucose. Indeed, it has been shown before that senescent CD4^+^ T cells exhibit an altered glucose energy metabolism by switching from glycolysis to the pentose phosphate pathway (Yang et al., 2016). Notably, young and old CD4^+^ T cells exhibited distinct capacities in response to the inhibition of glutaminolysis. Utilizing a Seahorse assay, we observed that the maximal respiratory capacity of mitochondria in old CD4^+^ T cells was limited. Moreover, when analyzing OCR and ECAR capacities simultaneously, mitochondrial respiration dropped, limiting the compensatory increase in glycolysis in old CD4^+^ T cells. Thus, limitations on maximizing metabolism or utilizing alternative energy pathways appear characteristic for the aging T‐cell metabolism. Those observations are supported by previous reports showing that several key components of the mitochondrial electron transportation chain and glycolysis pathway are defective in senescent cells (Gómez & Hagen, 2012; Weyand et al., 2014).

Indeed, when targeting glutaminolysis with DON, old CD4^+^ T cells exhibited a significant decrease in proliferation and effector function.

In contrast to conventional CD4^+^ T cells, Tregs functions had been specifically preserved in older recipients when inhibiting glutaminolysis. This observation is relevant as it distinguishes our metabolic approach from that of established immunosuppressants including calcineurin inhibitors that do not only inhibit T‐cell effector mechanisms but also interfere with the protective function of Tregs (De Serres et al., 2009). Thus, the selective immunosuppression of DON on effector CD4^+^ T cells may, at least in part, be linked to intrinsic metabolic phenotypes of T cells in aging that predominantly rely on lipid oxidation (Newton et al., 2016).

DON has also been effective in treating post‐transplantation cytomegalovirus infections (CMV) and de novo malignances (Chambers et al., 2010). Human CMV and a variety of other viruses use energy and molecular building blocks of infected cells to support replication (Rodriguez‐Sanchez et al., 2019). Of additional relevance, CMV‐infected cells augment glutamine uptake and glutaminase activity, emphasizing on the relevance of glutamine metabolism in CMV infections (Chambers et al., 2010). Moreover, modifications of glutaminolysis have also been recognized as a hallmark of cancer metabolism in recent years (Yang et al., 2017). The inhibition of glutaminolysis has also been shown to prevent primary tumor growth and systemic metastasis, establishing a role of DON as a cancer treatment (Lemberg et al., 2018; Shelton et al., 2010). Thus, at least in theory, DON may inhibit T‐cell‐mediated alloimmune responses while protecting from viral infections and neoplasm, aspects of relevance for immunosuppression in the elderly transplant recipient.

Metabolic inhibitors selectively target activated cells based on specific metabolic demands differing from those of naïve cells. Characteristically, most cell types have compromised metabolic demands in aging. Although not tested in sufficient detail, we assume that targeting glutaminolysis may be a feasible clinical approach in old recipients. Nevertheless, although we have not observed specific off‐target effects in our studies, effects of targeting glutaminolysis on other cells including intestinal epithelial cells and hematopoietic stem will need to be tested in more detail.

Of additional relevance are potential interactions between the inhibition of glutaminolysis and immunosuppressants (Fernandez‐Ramos et al., 2016). Calcineurin inhibitors, for example, reduce c‐Myc expression in murine CD4^+^ T cells through the inhibition of the NFAT/c‐Myc axis (Buchholz et al., 2006; Mognol et al., 2012). Moreover, CNIs can reduce the uptake of glucose and amino acids by increasing endocytosis and inhibiting transmembrane transports (Pereira et al., 2014; Sinclair et al., 2013). In addition, mTOR signaling pathways play an important role in the regulation of cellular metabolism (Fernandez‐Ramos et al., 2016), suggesting that the combinatorial application of agents targeting T‐cell metabolism and established immunosuppressants may display synergistic or additive effects.

In summary, our data demonstrate relevant changes in T‐cell metabolism in aging and introduce the inhibition of glutaminolysis as a novel age‐specific immunosuppression with relevance for but also beyond organ transplantation.

## 4 EXPERIMENTAL PROCEDURES

### 4.1 Antibodies and reagents

The following antibodies from eBiosciences were used for our flow cytometry analysis: anti‐CD4 (RM4‐5), anti‐CD8 (Ly‐3), anti‐IFN‐γ(XMG1.2), anti‐IL2(JES6‐5H4), anti‐IL17(eBio64CAP17), anti‐Foxp3 (FJK‐16s), and anti‐Thy1.1(OX‐7). Class II OVA peptides were obtained from AnaSpec. Rabbit polyclonal antibody to pS6 Ser 235/236 and c‐Myc was obtained from Cell Signaling. AntiCD3(17A2) and antiCD28(37.51) were obtained from BioLegend.

DON and Metformin were purchased from Sigma‐Aldrich. 2‐DG was obtained from Carbosynth. For all in vivo treatments, metabolic inhibitors were dissolved in PBS and administrated intraperitoneally.

### 4.2 Mice

Young (male, 8–12 weeks) and old (male, 18 months) mice C57BL/6 (H2b) were obtained from the National Institute of Aging (NIA, Bethesda, MD). Wild‐type DBA/2 (H2d; 8–12 weeks) mice were purchased from Charles River Laboratory (Wilmington, MA). OT‐II mice were purchased from Jackson Laboratory (Bar Harbor, ME). All animals were housed for 3 weeks in our facility prior to experiments; animals were allowed free access to water and standard chow. The use and care of animals were in accordance with National Institutes of Health and Institutional Animal Care and Use Committee guidelines. Experiments were approved by the Institutional Ethical Committee for Research on Animals.

### 4.3 Skin transplantation

Skin transplantation was performed using DBA donor mice and young or old C57BL/6 mice as recipients, representing a fully MHC mismatched donor/recipient combination served as allograft recipients. 1‐cm^2^ full‐thickness skin grafts were removed from the tail and engrafted onto the dorsolateral thoracic wall of recipients. Grafts were examined daily; graft rejection was defined as necrosis exceeding 90%.

### 4.4 Metabolic treatment

Skin transplant recipients received intraperitoneal injections of DON (1.6 mg/kg every other day) starting with the day of transplantation. Additional groups also received a triple metabolic inhibition consisting of DON (1.6 mg/kg DON every other day), metformin (150 mg/kg) and 2‐DG (0.5 g/kg).

### 4.5 Isolation of murine and human CD4^+^ cells

Single‐cell suspensions were obtained from mouse spleens and lymph nodes. Naïve CD4^+^ T cells were isolated by negative selection (Stemcell Technologies) using biotinylated antibodies directed against non‐CD4^+^ T cells (CD8, CD11b, CD11c, CD19, CD24, CD25, CD44, CD45R, CD49b, TCRγ/δ, TER119) and streptavidin‐coated magnetic particles.

Fresh peripheral blood mononuclear cells of healthy volunteers were isolated from heparinized blood using Ficoll‐Hypaque density centrifugation, and CD4^+^ T cells were isolated by negative selection according to the manufacture's protocol (Stemcell Technologies). The human study was approved by the Institutional Review Board (IRB) of Second Xiangya Hospital, Central South University. Informed consent was obtained from each volunteer in accordance with the Declaration of Helsinki. Cells were used at a purity of >95%; 6 samples were evaluated /group.

### 4.6 Cell culture

Splenocytes or T cells were cultured in RPMI 1640 media supplemented with 10% fetal calf serum, 200 mmol/L l‐glutamine, 100 U/ml penicillin/streptomycin, and 5 × 10^−5 ^mol/L b2‐mercaptoethanol at 37°C under a 5% CO_2_ and 95% air atmosphere with saturated humidity. For activation, CD4^+^ T cells were cultured in coated 96‐wells flat‐bottom plated with 10 µg/ml anti‐CD3 and 2 µg/ml soluble anti‐CD28 at a concentration of 1.5 × 10^6^ cells/ml.

### 4.7 Seahorse extracellular flux analysis

Twenty‐four hours after activation, 2 × 10^5^ naïve CD4^+^ T cells were harvested and resuspended with XF assay medium containing 25 mM glucose, 2 mM l‐glutamine, and 1 mM sodium. Cells were transferred to seahorse assay microplates and incubated in a non‐CO_2_ incubator at 37°C for 30 min. OCR and ECAR were measured at 37°C in an XF96 extracellular flux analyzer (Seahorse Bioscience) using manufacturer‐recommended protocols. After baseline measurements, OCR and ECAR were measured after sequentially adding metabolic inhibitors into each well according to manufacturer's instructions of Mito Stress assay or Mito fuel assay. Normalization of results was performed with CyQUANT assay (Invitrogen).

### 4.8 Flow cytometry

Cells were labeled for surface and intracellular antigens with fluorescence‐labeled antibodies. For intracellular staining, total splenocytes or isolated CD4^+^ T cells were seeded onto 96‐well plates and stimulated in complete media for 4 h at 37°C with phorbol 12‐myristate 13‐acetate (PMA; 50 ng/ml; Sigma‐Aldrich), ionomycin (500 ng/ml; Sigma‐Aldrich), and Brefeldin A (eBioscience). Thereafter, cells were fixed, permeabilized, and stained with respective antibodies at concentrations of 1–5 µg per 10^6^ cells. FOXP3, pS6 and c‐Myc staining were performed using manufacturer‐recommended protocols. Flow cytometry measurements were performed on a FACS Canto II (Becton Dickinson), and data were analyzed using FlowJo (FlowJo Software); gates were determined by comparison with unstimulated control cells.

### 4.9 Proliferation assays

In vitro proliferation of CD4^+^ T cells was determined by carboxyfluorescein succinimidyl ester (CFSE) dilution (eBioscience). Briefly, CFSE was dissolved in DMSO to a final stock solution of 10 mmol/L and then resuspended to 10^6^ cells/ml at a concentration of 1 μmol/L. Cells were incubated for 10 min at room temperature, then for 5 min. at 4°C and subsequently plated. For evaluation of proliferation in vivo, recipient mice were treated with DON or control solution (PBS) and received an intraperitoneal injection of 2 mg of bromodeoxyuridine (BrdU) solution (BD Biosciences) at the day of transplantation. Animals were euthanized by day 6; single‐cell suspensions of splenocytes were obtained and stained with APC‐labeled anti‐BrdU antibody. Fluorescence of CFSE‐ and BrdU‐labeled cells was assessed by flow cytometry as described in our previous publications (Krenzien et al., 2017; Quante et al., 2018).

### 4.10 CD8^+^ T‐cell depletion

Young and old C57BL/6 skin transplant recipients were treated with 100 µg anti‐mouse antiCD8 monoclonal purified antibody (Clone 53–6.7; BioLegend applied intraperitoneally) every other day starting with the day of transplantation. Treatment resulted in reliable depletion of >90% of CD8^+^ T cells in recipient mice as described in our previous work (Krenzien et al., 2017).

### 4.11 Adoptive transfers

CD4^+^ T cells were harvested from OT‐II mice, young or old wild‐type and purified by negative selection with Easysep CD4^+^ cell isolation kit. For OT‐II experiments, 1 × 10^6^ purified Thy1.1^+^CD4^+^ T cells were injected intravenously (retro‐orbital venous sinus) into Thy1.2^+^ C57BL/6 recipients. By 2 h, 500 μg OVA was administrated intraperitoneally triggering antigen‐specific responses. For adoptive transfer of young or old wild‐type CD4^+^ T cells, 2.5 × 10^6^ purified CD4^+^ T cells were injected intravenously on the day of transplantation.

### 4.12 ELISA

Cytokines were measured using commercial ELISA kits (eBioscience) according to the manufacturer's instructions.

### 4.13 Statistics

Kolmogorov–Smirnov and d'Agostino & Pearson omnibus normality tests were applied to verify Gaussian distribution. For parametric data, Student's *t* test or two‐sided one‐way ANOVA followed by Turkey's post‐test for comparing more than 2 groups was performed. For non‐parametric data, Mann–Whitney test or Kruskal–Wallis test followed by Dunnett's post‐test for comparing more than 2 groups was performed to test for statistical significance. Statistical significance for survival data was determined by log‐rank Mantel–Cox test. The level of significance was chosen to be at *p* < 0.05. All statistical analyses were calculated with GraphPad Prism (version 7).

## CONFLICT OF INTEREST

The authors declare no competing interest.

## AUTHOR CONTRIBUTIONS

Y.N., J.I., and R.M. performed experiments, analyzed data, and wrote the manuscript. K.M. and T.H. performed experiments. H.A., Y.L., J.Y., R.A., and HZ supported experiments and edited the manuscript. A.E. and S.G.T. designed experiments, supervised the work, and wrote the manuscript.

## Supporting information

Fig S1

Fig S2

Legends S1‐S2

## Data Availability

There are no Protein, DNA, RNA Sequences/Sequences of RNAi, antisense, and morpholino probes/Human Genomic Data Reporting Newly Described SNPs and CNVs Identified in Control/Human Sequence Data/Microarray Data and Structures of Small Molecules included in this study. All other data that support the findings of this study are available on request from the corresponding author (SGT).
