# Supplementary figures and images for: Targeting age‐specific changes in CD4+ T cell metabolism ameliorates alloimmune responses and prolongs graft survival
Source: Aging Cell. 2021 Jan 26;20(2):e13299. doi: 10.1111/acel.13299 (PMC7884034; doi:10.1111/acel.13299)

A.

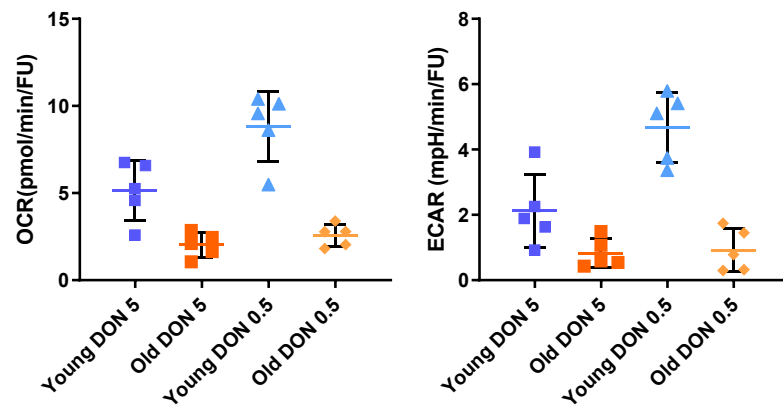

B.

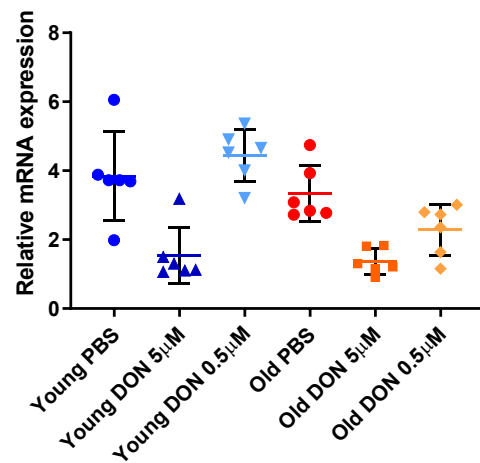

Supplement: Supplementary file 1 — Fig S1 [file ACEL-20-e13299-s001.pdf]
