# Supplementary material for: Targeting age‐specific changes in CD4+ T cell metabolism ameliorates alloimmune responses and prolongs graft survival
Source: Aging Cell. 2021 Jan 26;20(2):e13299. doi: 10.1111/acel.13299 (PMC7884034; doi:10.1111/acel.13299)
Supplement: Supplementary file 2 — Fig S2 [file ACEL-20-e13299-s002.pdf]

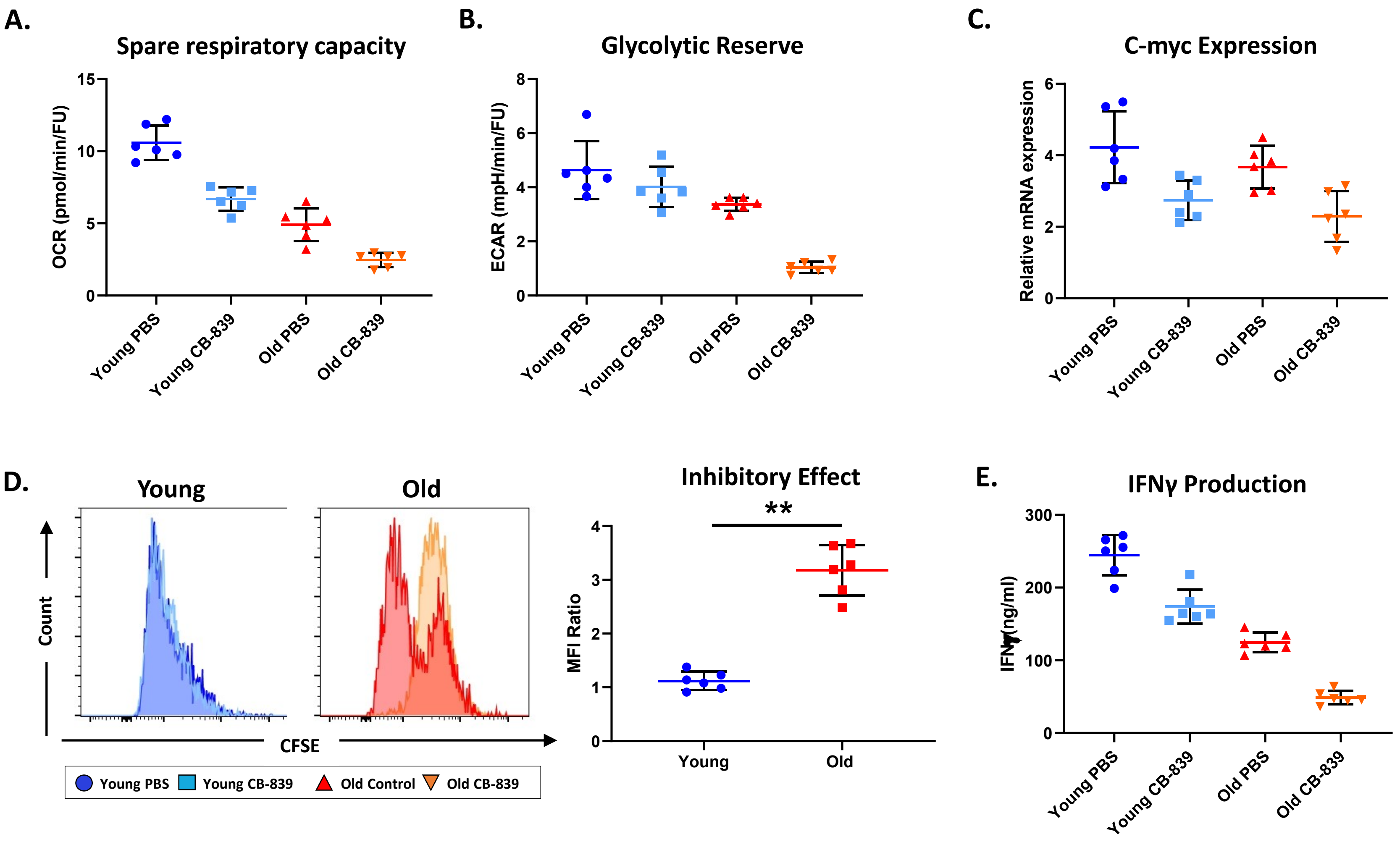

A.
Spare respiratory capacity
OCR (pmol/min/FU)
15
10
5
0
Young PBS
Young CB-839
Old PBS
Old CB-839
B.
Glycolytic Reserve
ECAR (mpH/min/FU)
8
6
4
2
0
Young PBS
Young CB-839
Old PBS
Old CB-839
C.
C-myc Expression
Relative mRNA expression
6
4
2
0
Young PBS
Young CB-839
Old PBS
Old CB-839
D.
Young
Old
Count
CFSE
Young PBS
Young CB-839
Old Control
Old CB-839
Inhibitory Effect
**
MFI Ratio
4
3
2
1
0
Young
Old
E.
IFNγ Production
IFNγ (ng/ml)
300
200
100
0
Young PBS
Young CB-839
Old PBS
Old CB-839
