# Supplementary material for: Targeting age‐specific changes in CD4+ T cell metabolism ameliorates alloimmune responses and prolongs graft survival
Source: Aging Cell. 2021 Jan 26;20(2):e13299. doi: 10.1111/acel.13299 (PMC7884034; doi:10.1111/acel.13299)
Supplement: Supplementary file 3 — Legends S1‐S2 [file ACEL-20-e13299-s003.docx]

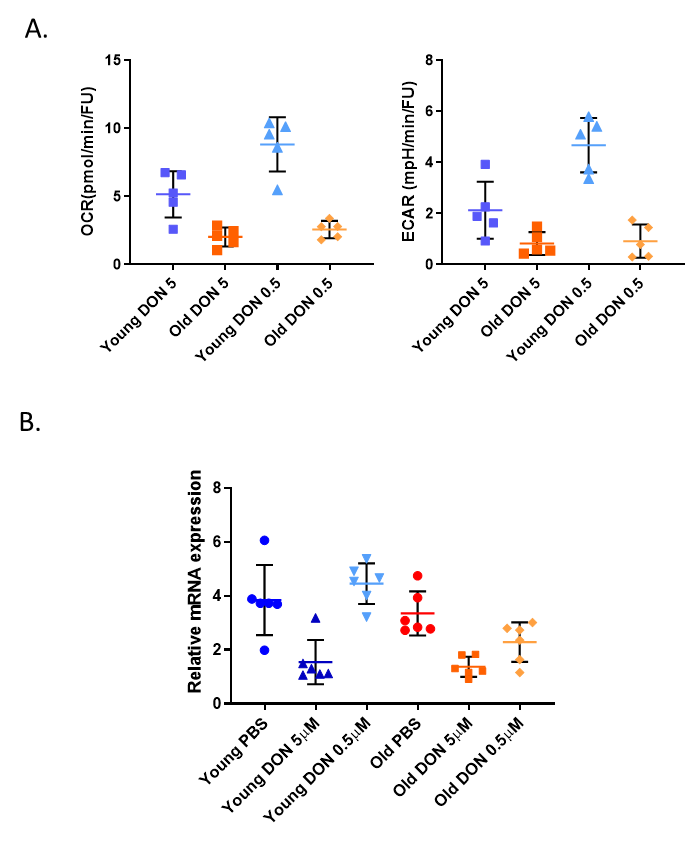


**Supplement Figure 1**

**(A)** OCR and ECAR of young and old naïve CD4^+^ T cells were measured by 24 hrs after anti-CD3/anti-CD28 stimulation with DON (5 μM, 0.5 μM or PBS). Control group were maintained in 50 ng/ml IL-2. Difference in OCR and ECAR between young and old naïve CD4^+^ T cells was compared. Column plots display individual data points and mean±SD, n=5/group. **(B)** c-Myc expression were measured by RT-PCR in young and old naïve CD4^+^ T cells in presence of DON (5 μM, 0.5 μM) or PBS. Column plots display individual data points and mean±SD, n=6/group. The results are representative of at least three independent experiments.


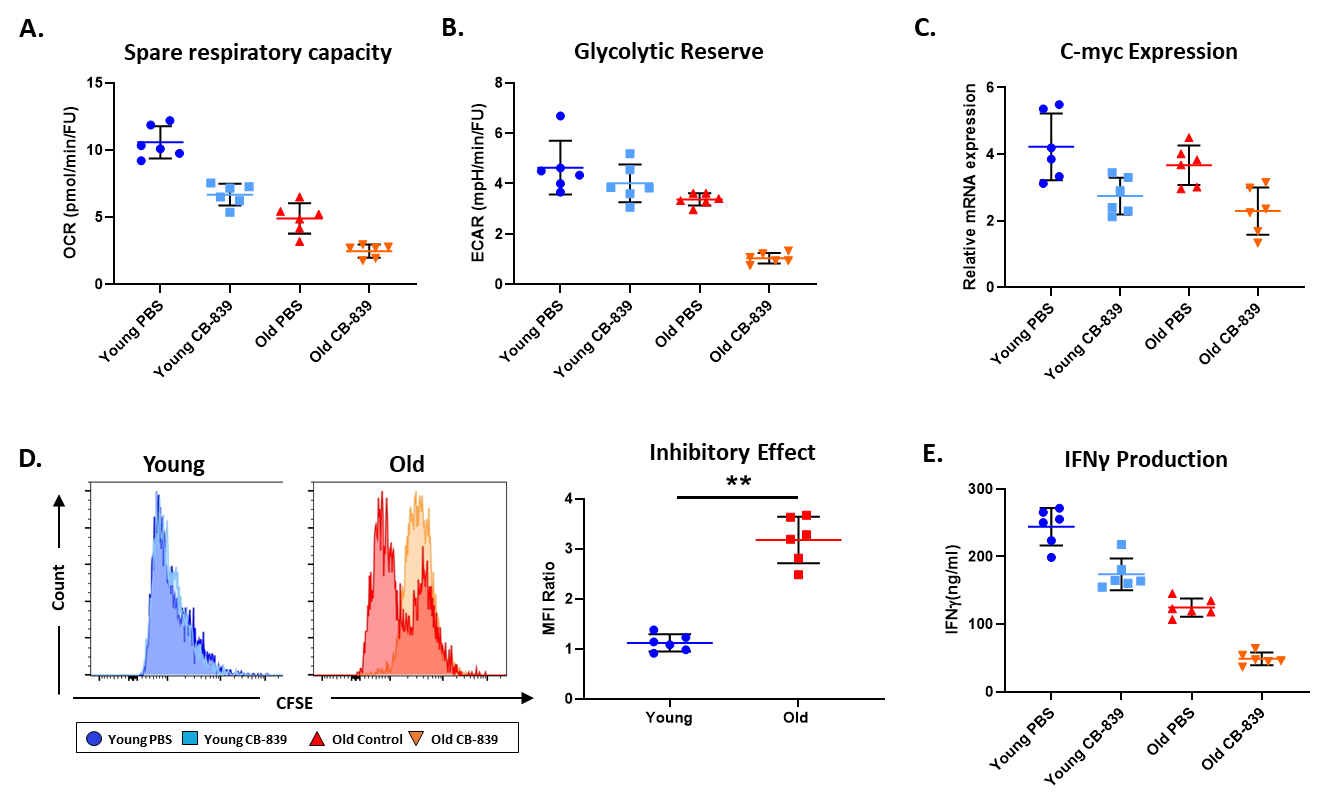


**Supplementary Figure 2. Age-Specifc immunosuppressive effects of DON are not mediated through CTPS1 inhibition**

**(A and B)** Naïve CD4^+^ T cells were isolated from young and old C57BL/6 mice and activated with 10 µg /mL anti-CD3 and 2 µg /mL soluble anti-CD28 for 24 hours. Calculated spare respiratory capacity and glycolytic reserve were assessed utilizing a Seahorse Mito-stress. **(C)** C-myc **(D)** Naïve CD4^+^ T cells were isolated from young and old C57BL/6 mice, cultured in a mixed lymphocyte reaction with DBA-derived splenocytes in addition of either PBS or CB-839 (1μM) labeled with CFSE. After 72 hrs, proliferation of naïve CD4^+^ T cells was measured by the dilution of CFSE and the inhibitory effects of CB-839 measured by the ratio of CFSE median fluorescence intensity between the CB-839 and the PBS group.

Column plots display individual data points and mean±SD, n=5/group. Statistical significance was determined by using Mann-Whitney test. Asterisks indicate p-values * = p≤0.05, **= p≤0.01 and *** = p≤0.001, only significant values are shown. The results are representative of at least three independent experiments.
